# Pleasures of the Mind: What Makes Jokes and Insight Problems Enjoyable

**DOI:** 10.3389/fpsyg.2017.02297

**Published:** 2018-01-24

**Authors:** Carla Canestrari, Erika Branchini, Ivana Bianchi, Ugo Savardi, Roberto Burro

**Affiliations:** ^1^Department of Education, Cultural Heritage and Tourism, University of Macerata, Macerata, Italy; ^2^Department of Human Sciences, University of Verona, Verona, Italy; ^3^Section Philosophy and Human Sciences, Department of Humanities, University of Macerata, Macerata, Italy

**Keywords:** pleasures of the mind, humor, cartoons, insight problem solving, the “Aha!” experience, enjoyability

## Abstract

In this paper, a parallel analysis of the enjoyment derived from humor and insight problem solving is presented with reference to a “general” Theory of the Pleasures of the Mind (TPM) (Kubovy, 1999) rather than to “local” theories regarding what makes humor and insight problem solving enjoyable. The similarity of these two cognitive activities has already been discussed in previous literature in terms of the cognitive mechanisms which underpin getting a joke or having an insight experience in a problem solving task. The paper explores whether we can learn something new about the similarities and differences between humor and problem solving by means of an investigation of what makes them pleasurable. In the first part of the paper, the framework for this joint analysis is set. Two descriptive studies are then presented in which the participants were asked to report on their experiences relating to solving visuo-spatial insight problems (Study 1) or understanding cartoons (Study 2) in terms of whether they were enjoyable or otherwise. In both studies, the responses were analyzed with reference to a set of categories inspired by the TPM. The results of Study 1 demonstrate that finding the solution to a problem is associated with a positive evaluation, and the most frequent explanations for this were reported as being Curiosity, Virtuosity and Violation of expectations. The results of Study 2 suggest that understanding a joke (Joy of verification) and being surprised by it (Feeling of surprise) were two essential conditions: when they were not present, the cartoons were perceived as not enjoyable. However, this was not enough to explain the motivations for the choice of the most enjoyable cartoons. Recognizing a Violation of expectations and experiencing a Diminishment in the cleverness or awareness initially attributed to the characters in the cartoon were the aspects which were most frequently indicated by the participants to explain why they enjoyed the joke. These findings are evaluated in the final discussion, together with their limitations and potential future developments.

## Introduction

Everyone would immediately agree that humor belongs to the category of pleasurable human activities. The majority of experimental work on humor has focused on appreciation (which is clearly related to pleasure), and various theories regarding the pleasure we get from humor have been put forward. However, there are still new aspects of this topic to investigate, and this paper explores one of these by means of a comparison between the sensations of pleasure triggered in two different but related cognitive activities: humor and insight problem solving.

The processes which are activated in insight problem solving have many structural features which also relate to humor, for example, puzzlement, instantaneous understanding, surprise, a collision of contrasting cognitive schemas and a subsequent representational change to overcome this contrast (e.g., Gick and Lockhart, 1995; Kozbelt and Nishioka, 2010; Korovkin and Nikiforova, 2015). Parallels between what happens when people “get” a joke and when they successfully solve an insight problem have been already made from a number of different perspectives (e.g., Schiller, 1938; Koestler, 1964; Suls, 1972, 1983; Fagen, 1981; Pepiciello, 1989; O'Quin and Derks, 1997; Derks et al., 1998). In both cases there is a kind of conundrum which needs to be resolved. A conundrum in the case of humor, such as in a joke for instance, often involves an incongruity in the punch line. When the joke is understood, this incongruity is resolved and a feeling of satisfaction, and therefore pleasure may arise. In insight problem solving too, there is typically a conundrum which may be either visual or verbal (Dominowski and Dallob, 1995; Öllinger and Knoblich, 2009).

What occurs in both cases is that the problem solver suddenly realizes that a representational change needs to be made in order for the incongruity to be resolved. This change requires a shift outside the initial representation of the problem (Ohlsson, 1992; Knoblich et al., 1999, 2001; Öllinger et al., 2006, 2008). Instantaneous understanding (Kozbelt and Nishioka, 2010, p. 377) and a fairly automatic revision or reorganization of the initial representation (Gick and Lockhart, 1995, p. 224) are therefore two of the basic features of the restructuring process that are common to both understanding humor and solving insight problems.

As a result of this similarity, some studies have even addressed the issue of whether humor might function as a facilitator in insight problem solving (Gick and Lockhart, 1995; Martin, 2007; Kozbelt and Nishioka, 2010; Korovkin and Nikiforova, 2015). The rationale for this, as identified by some researchers, relates to attentional processes, that is humor relieves stress thereby diluting the degree of attention being devoted to the problem (Rowe et al., 2007). This in turn stimulates the problem solver's “peripheral focus,” destabilizing perceptual and thought patterns and producing a positive effect in terms of overcoming fixities and helping people to change their perspective in order to restructure the problem (Korovkin and Nikiforova, 2015). It has also been argued that humor strongly promotes associative thinking, in particular stimulating remoteness of association and the creation of non-obvious connections (Koestler, 1964; Goodchilds, 1972; Besemer and Treffinger, 1981; Sitton and Pierce, 2004). These are all related to creativity (Mednick, 1962; Koestler, 1964; Ellwood et al., 2009; Gilhooly et al., 2012, 2013) and have a facilitatory effect in insight problem solving where the solution cannot be reached by simply reproducing familiar procedures. Creative or divergent processes are required (Dominowski and Dallob, 1995; Öllinger and Knoblich, 2009).

Whereas various studies have analyzed the points of convergence relating to the cognitive processes involved in both humor and problem solving, very little research has been done into whether humor and problem solving also share points of convergence relating to the pleasurable emotions they elicit (Schiller, 1938; Csikszentmihalyi, 1990; Kahneman et al., 1999; Kubovy, 1999), despite evidence that both activities imply associative thinking and are frequently accompanied by positive emotions and moods (Schiller, 1938; Bar, 2009; Korovkin and Nikiforova, 2010; Brunyé et al., 2013; Trapp et al., 2015). The present paper aims to explore this topic further by analyzing both humor and problem solving using the same conceptual tool. The basis for this tool is a general Theory of the Pleasures of the Mind (TPM) that was published by Kubovy (1999) in a book edited by the Nobel prize winner Kahneman in collaboration with Diener and Schwarz. The subject of the book regards a complex and challenging topic, *Well-being: the foundations of Hedonic Psychology* (1999). In the various chapters forming this book, the contributors address the puzzle of what humans like and dislike, within the mindset of experimental science. In the set of empirical evidence used by Kubovy to support his theory, the relationship between humor and problem solving is hinted at but not focused on in detail. Providing experimental evidence concerning the grounds of this relationship, however, might provide a significant contribution toward a further development of the TPM. This paper aims to delve into this connection, on the one hand by strengthening any evidence resulting from a comparison of the literature on these two cognitive activities and on the other hand by proposing an empirical paradigm in order to explore this relationship experimentally.

In section Placing the Pleasure Elicited by Humor and Insight Problem Solving within a General Research Framework for Exploring Pleasures of the Mind we will briefly present the TPM and outline the reasons why it has been chosen as a point of reference. We will then discuss how in our view this “general” perspective is connected to more “local” approaches, that is, approaches that have been developed specifically to study the enjoyment people derive from humor (section Connections between the TPM Approach and More “Local” Theories on Humor) or from insight problem solving (section Connections between the TPM Approach and More “Local” Theories Relating to the Emotions Elicited by Insight Problem Solving). In the second part of the paper, we present two descriptive studies (sections Study 1: Factors Determining Enjoyment and Lack of Enjoyment in Insight Problem Solving and Study 2: Factors Determining Enjoyment or Lack of Enjoyment in Humor) that were carried out with a two-fold aim: first, to explore the applicability of the common categories of the TPM in terms of operationalizing the enjoyment (or lack of enjoyment) relating to tasks involving visuo-spatial insight problem solving (Study 1) and to humorous cartoons (Study 2), and second, to ascertain whether the results of these two studies reveal any potential benefits of using the same operational categories to investigate these topics.

## Placing the pleasure elicited by humor and insight problem solving within a general research framework for exploring pleasures of the mind

Whereas it is fairly evident that people experience humor as a pleasant experience, it is less obvious how this construct can be operationalized. This type of pleasurable feeling has been referred to in terms of amusement, appreciation, mirth, exhilaration, cheerfulness, hilarity, merriment and even sudden glory (e.g., Zweyer et al., 2004; Martin, 2007). All these facets of what is in effect a generally complex construct can, taken individually, be empirically investigated (e.g., Ruch et al., 1996, 1997; for an overview see Ruch, 1998).

Kubovy (1999) discussed humor as an example of a pleasurable experience within a different theoretical framework, i.e., one which aims to define the universals of pleasurable intellectual experiences such as, for example, listening to music, reading poetry, solving puzzles, bird watching, and gardening. This general theory is not usually mentioned in the literature on humor, but it seems to us to represent a comprehensive approach which encompasses the perspectives on pleasure derived from humor which have been, more or less explicitly, developed elsewhere in mainstream approaches to the subject (e.g., Keith-Spiegel, 1972; Martin, 2007; Larkin-Galiñanes, 2017).

According to the TPM, there are three main notions which go toward defining the concept of “pleasure of the mind”: (1) the stimuli and activities that induce pleasures of the mind give rise to certain patterned sequences of emotions; (2) a feeling of satisfaction occurs when a definite set of expectations (the so-called *prior state*) is violated (the *onset* moment), thereby triggering a search for an interpretation (i.e., *change*) which in turn leads to the resolution of a situation or problem, and (3) there are a number of emotions that are present to varying degrees in most pleasures of the mind (*curiosity, feeling of surprise, joy of verification, virtuosity*, and *diminishment*). This harks back to Scheffler's (1991) definition of cognitive emotions as emotions that rest on a supposition relating to the contents of a person's propositional attitudes (beliefs, predictions, expectations) and bear on its epistemological status (e.g., confirmation).

More specifically, pleasures of the mind are defined as a collection of emotions distributed over time (Kubovy, 1999, see also Kahneman, 1988, 1999). The basic structure of a pleasurable episode (or stimulus) comprises an initial set of kernels that elicits a *prior state* (i.e., a set of expectations and interpretations related to the episode), a following set of kernels (i.e., *onset*) that produces a violation of the prior state triggering a search for a new interpretation (i.e., *change*) of the initial set of kernels. The emotions associated with this sequence are *suspense* (at the onset stage), which can be accompanied by *fear* or *hope* and *automatic nervous system arousal* due to the violation of expectations. At this point, *curiosity*, that originates from the unknown, emerges and triggers a search for a new interpretation. When a decision on how to reconstruct the initial interpretation has been made at the change stage, various emotions arise: feelings of *surprise*, due to the switch from the initial set of interpretations to the final one; *joy* in verifying the aptness of the new interpretation; *satisfaction* with performing a new skill (i.e., feeling virtuous due to success in finding a new interpretation) and sometimes *superiority* on discovering that the new interpretation produces a diminishment of the value of the initial interpretation. The sensation of suspense which produces tension due to the inadequacy of the initial interpretation gives way to a final feeling of relief.

Kubovy (1999, p. 146) suggests that this analysis can also apply to humor and hints at the fact that it might apply to problem solving too. We used this as a starting point to our investigation.

### Connections between the TPM approach and more “local” theories on humor

We carried out a detailed analysis of the emotions that, according to various studies and theories, are said to be sequentially elicited by humor, going beyond the references mentioned by Kubovy (1999) in his original paper. We found that the TPM is in fact consistent with the core concepts of the three main approaches to humor and it somehow unites them. These are: the cognitive approach (i.e., the incongruity-resolution theory); the psycho-physiological approach (i.e., the release theory); and the sociological approach (i.e., superiority and disparagement theories). If we consider how the TPM applies to the pleasure associated with hearing a good joke, we can understand how this works. The final part of a joke, that is, the punch line, often produces a sudden and unexpected incongruity (Suls, 1972) since it is not coherent with the preceding phase (usually called the set up) and with the expectations, predictions, interpretations which have been established as part of the set up (i.e., the *prior state* in the TPM). This incongruity (referred to as the *onset* in the TPM) elicits a specific feeling referred to as, variously, confusion of thought (Maier, 1932, p. 70), puzzlement (Schiller, 1938; Berlyne, 1972, p. 56) and embarrassment (Schiller, 1938). The violation of the *prior state* provoked by the punch line triggers a *change* in the interpretation of the initial kernel on which the prior state is based (according to the TPM), and this is consistent with what both cognitive approaches to humor (e.g., Koestler, 1964; Suls, 1972; Attardo and Raskin, 1991; Giora, 1991; Vaid et al., 2002; Forabosco, 2008) and comprehensive theories of humor would claim (e.g., Apter, 1982; Wyer and Collins, 1992; Attardo, 2017). With reference to the former, in particular, this change in interpretation is the result of the resolution of the incongruity. It has also been demonstrated that this pattern elicits pleasurable emotions in those who are telling the joke (Hull et al., 2016).

Leaving aside the structure of the kernels, let us now focus on the emotions that, according to the TPM, are produced by and typically characterize pleasurable experiences in order to determine whether studies on the enjoyment that people derive from jokes also identified the same specific sensations.

- *Curiosity*—the pleasure which comes from satisfying curiosity, that is, learning something new, involves a shift from an epistemic stance of the unknown or the uncertain to the known. This is something which has been identified as often characterizing people's experience of humor (Watts, 1989; Canestrari et al., 2014).
- *Feeling of surprise*—various authors have emphasized that feeling surprised is a necessary condition for humor to be a pleasing experience although it is not the only necessary condition (e.g., Maier, 1932; Suls, 1972; for an overview of the early conceptions of surprise theories relating to humor, see Keith-Spiegel, 1972); with specific reference to jokes, the feeling of surprise has been operationalized in terms of an *optimal innovation*, that is, a pleasing balance between novelty and salience (Giora, 2002).
- *Joy of verification*—according to the TPM, this emotion typically occurs when people find the solution to a problem and it can be argued that this also applies to the processes related to understanding a joke when an incongruity is resolved. However, in cognitive literature on the resolution of incongruities in humor, it has often been pointed out that this resolution is incomplete since a residual incongruity persists even after the listener or reader “gets” the joke (e.g., Koestler, 1964; Rothbart and Pien, 1977; Apter, 1982; Ziv, 1984; Forabosco, 1992; Wyer and Collins, 1992). This may constitute a structural difference between problem solving and understanding humor: in the former case (i.e., when there is a “serious” incongruity), the resolution renders the initially problematic elements of the situation completely coherent with the solution, without any “residuals”; this does not apply to humor in which case a radical re-interpretation of the *prior state* is implied without the implications of the initial interpretation being eliminated. This has been described by, for example, Apter (1982) in terms of the simultaneous perception of two contradictory and synergetic viewpoints. Beattie also observed that “*an uncommon mixture of relation and contrariety, exhibited, or supposed to be united, in the same assemblage*” provokes a pleasant emotion whose external sign is laughter (Beattie, 1776, p. 454) and Koestler referred to the bisociation theory as a key element of humor, that is, “*the perceiving of a situation or idea […] in two self-consistent but habitually incompatible frames of reference*” (1964, p. 35, italics by the author).
- *Virtuosity*—virtuosity seems to fit in well with the idea of *cognitive mastery* (e.g., McGhee, 1974; Forabosco, 1992, 2008); in the context of humor, the term cognitive mastery refers to the cognitive competence required to make an incongruity congruous; the acquisition and evolution of this ability depend on cognitive development (e.g., McGhee, 1974; Forabosco, 2008), for example, the humorous incongruities which can be understood in early childhood are very simple (such as a funny face), but more complex forms of incongruity (for example irony) are only understood much later (Dews et al., 1995; Pexman et al., 2005; Angeleri and Airenti, 2014; Bianchi et al., 2017); the term virtuosity, which in the TPM refers to the pleasure derived from doing something that we could not do before (Kubovy, 1999, p. 147), is clearly applicable to this sense of mastery signaling a cognitive development (e.g., McGhee, 1979; Pien and Rothbart, 1980; Bergen, 1998), something which is in fact frequently impaired in a number of mental disabilities (e.g., Forabosco, 1998, 2008; Ivanova et al., 2014).
- *Violation of expectations*—in the TPM the sense of relief comes from a relaxation of the initial tension caused by a violation of the expectations established in the prior state; the incongruity which arises between the initial interpretation of the joke and the punch line results in a feeling of tension or suspense which is only relieved when the joke is understood; this is consistent with the psycho-physiological approach to humor which goes back to Spencer and Freud and was also later developed in relation to humor in art works (e.g., Berlyne, 1972; Wyer and Collins, 1992; Bonaiuto, 2006); Berlyne (1972) explicitly connects the feelings of confusion or tension elicited by the perception of an incongruity to the hedonic value of humorous stimuli since they result in an increase in arousal which is released when the incongruity is resolved and the humor is understood;
- *Diminishment—*in the TPM, and according to the theories developed by Wyer and Collins (1992) and Apter (1982), it is possible that a reinterpretation of the kernel may diminish some aspects of the initial interpretation thereby eliciting a feeling of superiority. This is in line with superiority theories which claim that humor often involves laughing at someone else's weakness, defect, or misfortune (for a review see Keith-Spiegel, 1972; Martin, 2007; Larkin-Galiñanes, 2017).

### Connections between the TPM approach and more “local” theories relating to the emotions elicited by insight problem solving

The Eureka moment or “Aha!” experience, that is the moment in which the solution pops up in problem solvers' minds, suddenly and unexpectedly (Durso et al., 1994; Wegner, 2002), can be regarded as the defining feature of insight. Studies aiming to describe the insight experience focused on the “Aha!” experience (Kaplan and Simon, 1990; Gick and Lockhart, 1995; Bowden and Jung-Beeman, 1998; Boden, 2004; Bowden et al., 2005; Kounios et al., 2006; Danek et al., 2013, 2014a,b; Fedor et al., 2015; Hedne et al., 2016; Salvi et al., 2016; Shen et al., 2016; Webb et al., 2016). It has been demonstrated that the “Aha!” experience is not a unitary construct but a multidimensional one in which there is an interplay of cognitive and emotional components. Some of these components map with the emotions that, according to the TPM approach, characterize pleasurable events in general (and also specifically humor).

- *Curiosity*, according to the TPM is characterized by an initial state of tension related to not knowing something and by a final state of relief when the new information is acquired. Danek et al. (2014b) stated that “the release of tension” is in fact an aspect characterizing the “Aha!” experience. In insight problems, tension arises from the very beginning, since there is no obvious solution to the problem, and unsuccessful problem solving attempts built the tension up further. If finally a solution is found, the tension rapidly declines. Drive, that is another aspect of the “Aha!” experience which consists of the motivation to work and to continue working on the problem (Ohlsson, 1984; Danek et al., 2014a,b), also belongs to this category.
- *Feeling of surprise*, in problem solving, is associated with the disclosure of the solution. It has been proved that it can vary in strength, and it can be accompanied by either positive (delight) or negative (chagrin) emotions (Gick and Lockhart, 1995; Danek et al., 2014a,b; Hill and Kemp, 2016).
- *Joy of verification* corresponds to what, in the literature on problem solving, has been called the “intuitive sense of success,” that is, the certainty that an insightful solution is correct (Gick and Lockhart, 1995; Bowden and Jung-Beeman, 2007; Danek and Wiley, 2017). This aspect has also often been described in relation to scientific discoveries (Gick and Lockhart, 1995).
- *Virtuosity* captures the emotions that in the literature on problem solving have been referred to as “performance-related aspects” (Danek et al., 2014b), which are manifested in the problem solvers' comments about their ability to find the solution to a problem.
- *Violation of expectations* corresponds to what has been described as suddenly realizing that certain features which were not obviously relevant, and in fact were not initially focused on and encoded (“selective elaboration” or “selective encoding,” Ohlsson, 1984; Gick and Lockhart, 1995; Danek et al., 2014b), were in reality extremely relevant to the solution.
- *Dimishment* has been not mentioned as such in previous literature on problem solving, but it may be applied to the emotion of discovering that the solution to the problem was right under the problem solver's eyes but hidden (Dominowski and Dallob, 1995; Öllinger and Knoblich, 2009). This emotion has been conceptualized and discussed more in terms of negative than of positive emotions, i.e., a sort of chagrin due to the problem solvers' prior stupidity (Gick and Lockhart, 1995; Danek et al., 2014b; Hill and Kemp, 2016).

## Study 1: factors determining enjoyment and lack of enjoyment in insight problem solving

In the previous section (section Connections between the TPM Approach and More “Local” Theories Relating to the Emotions Elicited by Insight Problem Solving), it was shown that the cognitive emotions referred to in the TPM are not extraneous to the emotions revealed in other studies on insight problem solving. We might also ask whether they constitute a systematic list to usefully support empirical investigations into self-reports from problem solvers.

In this study, we focused on visuo-spatial insight problems. Three different conditions were investigated. These differed in terms of the degree of direct engagement of the problem solver in the search for a solution: in a relatively “standard” condition, the participants were given 7 min to solve each problem (e.g., Schooler et al., 1993; Fleck and Weisberg, 2013; Ball et al., 2015); in another condition, the time at their disposal was reduced to 3 min, and, in the third condition, the participants were not asked to try to solve the problems, but were instead immediately given a sheet of paper showing the solutions. In all of the conditions which were tested, after the solutions were revealed, the participants were asked to indicate which two problems they liked the most, which two they liked the least, and to explain their choices. Their explanations were analyzed in terms of a set of categories which had been derived from the TPM and re-formulated as “operational categories” (see Table 1). This is a descriptive study. There were no specific expectations regarding how frequently the various different categories would occur and there were no precise predictions about whether successfully solving the problems (or not solving them) would have a linear effect on the motivations the participants gave for why they found the problems enjoyable or not. We were rather aiming to explore whether analyzing responses in terms of these categories would lead to a meaningful pattern which might in turn indicate a further predictive research phase.

**Table 1:** The operational categories used to analyze the explanations provided by participants in Study 1.

Curiosity	***The TPM:*** Being curious means that you get pleasure from learning something that you did not previously know. ***Definition in Problem Solving:*** Curiosity is experienced by problem solvers when, in the initial stage (problem setting), they feel a state of tension related to not knowing the solution. It is the experience of “missing something” (the solution) that prompts them to look for what they do not know yet (i.e., to move from the unknown to the known). When they know the solution, they know something new and this leads to a final state of relief. ***Examples (most enjoyable problems)***: “I was very intrigued by the problem… and then also by the solution” [pigs in a pen]; “This was the problem that from the beginning most roused my curiosity” [triangle]. ***Examples (least enjoyable problems)***: “I did not find the solution and I felt as if I was left up in the air, out of the picture” [pigs in a pen]; “The problem did not make me curious, even after I learned what the solution was” [five-square].
Virtuosity	***The TPM:*** Virtuosity refers to the pleasure you have when you feel you are doing something well. ***Definition in Problem Solving*****:** Virtuosity relates to experiencing the mastery of being able to cope with the task, to reason about possible solution paths. When problem solvers find the correct solution, they feel proud of their reasoning skills. (note: both virtuosity and curiosity concern stepping from an initial state of not knowing the solution—and not knowing whether one will be able to discover it – to knowing it. However, the focus in curiosity is on “learning a new content,” whereas in virtuosity the focus is on discovering, or confirming, one's reasoning skills). ***Examples (most enjoyable problems)***: “The kind of reasoning involved was both intuitive and mathematical and presupposed a bit of knowledge of the subject” [circumference]; “It was thought provoking in terms of the reasoning which it necessitated” [eight-coins]. ***Examples (least enjoyable problems)***: “I remained focused for too long on the lines forming the head [in the deer problem] and my brain got stuck; then it was impossible to start reasoning in other directions”; “In order to solve this problem [the circumference problem] you needed to apply geometrical rules that you were supposed to remember.”
Violation of expectations	***The TPM:*** You search for an interpretation of the source of the violation of your expectation. You get pleasure from the violation of expectations followed by a return to a stable state. ***Definition in Problem Solving*****:** A violation of expectations is experienced by problem solvers when they realize that a change in their initial mental representation of a problem is needed (since it is misleading) and that information that has been viewed as insignificant is in reality relevant to the solution (i.e., a shift in the focus of attention). ***Examples (most enjoyable problems)**:* “The solution leads us away from the usual way of thinking because we are used to thinking of a square as being oriented with two vertical and two horizontal lines while the solution requires them to be oblique” [pigs in a pen]; “I focused from the beginning on moving the lines representing the head and only those. I never thought of moving the legs!” [deer]. ***Examples (least enjoyable problems)**:* “The position that the deer has in the solution (sitting that way!) is not a normal position” [deer]; “I thought we had to move the coins only on the plane, while the solution was to put some on top of others” [eight coins].
Feeling of surprise	***The TPM:*** This emotion is familiar to scientists but is widespread in entertainment as well. It is a feature much sought after in the mystery genre. ***Definition in Problem Solving*****:** The experience of surprise (positive surprise, negative surprise or no surprise) is associated with the disclosure of the solution. (note: Feeling of surprise is associated with unexpectedness, and is therefore often likely a consequence of the problem solvers' expectations being violated—see previous category. When, in order to explain their choices, participants explicitly referred to their expectations as being violated, responses were classified in the previous category. When they simply referred to the amazement (positive) or perplexity or no feeling of surprise (both negative) that they experienced when the solution was revealed, responses were classified in the present category). ***Examples (enjoyable problems)***: “The solution surprised me” [deer]; “The solution astonished me” [pigs in a pen]. ***Examples (least enjoyable problems)***: “The solution did not surprise me” [five square]; “The correct solution surprised me negatively: I found it meaningless”[eight coins].
Joy of verification	***The TPM:*** This emotion is familiar to scientists but is widespread in entertainment as well. The joy of verification is a characteristic of many puzzles. ***Definition in Problem Solving*****:** Joy of verification is experienced by problem solvers in terms of proximity to the correct solution. (note: this category differs from Virtuosity in that participants do not explicitly refer to the pleasure of the reasoning acts they were engaged in but to their experience of verifying that their solution was in fact the correct one—or close to it). ***Examples (most enjoyable problems)***: When I received the response sheet, I verified that my solution was the right one” [circumference]; “I came very very close to the correct solution” [deer]. ***Examples (least enjoyable problems)***: “I was close but still wrong” [deer]; “When I was given the solution, I realized I was very far away from the correct solution” [triangle].
Diminishment	***The TPM:*** If the reinterpretation paints a less desirable picture of the protagonist or the event (…), then you will find the event to be humorous. ***Definition in Problem Solving*****:** Diminishment in problem solving is associated with a person realizing that the solution was simple while they had been trying much more complex reasoning paths. This can lead to enjoyment when the person makes fun of his/her own too convoluted reasoning (i.e., diminishes him/herself) or can lead to negative feelings when the person diminishes the problem to the status of a trivial one. ***Examples (most enjoyable problems)***: “When I read the solution, I found it so interestingly simple” [pigs in a pen]; “The solution was so simple and obvious, but at the same time very clever” [deer]. ***Examples (least enjoyable problems)***: “It was a very easy problem in the end” [circumference problem]; “The problem was too banal and the solution too elementary” [triangle].
Happiness	***Definition in Problem Solving***: A pure expression of amusement and/or enjoyment without any specific explanation for its cause. ***Examples (most enjoyable problems)***: “I liked it from the very beginning” [triangle]; “I found the solution nice” [deer]. ***Examples (least enjoyable problems)***: “Even after I learned the solution, I did not like it” [five-square]; “I do not like this problem” [eight coins].
Content type	***Definition in Problem Solving*****:** An expression of amusement and pleasure related to the specific *kind* of process that needs to be activated in order to search for a solution, independently of feeling able to do it or not. ***Examples (most enjoyable problems)***: “I always enjoy working on problems with non-geometrical figures” [pigs in a pen] ; “I adore puzzles that require me to pay great attention to the words used in the text” [eight coins]. ***Examples (least enjoyable problems)***: “I don't like Geometry” [circumference]; “I have never liked solving Geometry problems” [circumference].
Superficial aspects	***Definition in Problem Solving*****:** An expression of amusement and pleasure related to the superficial elements of the problem or to the images depicted in the problem. ***Examples (most enjoyable problems)***: “The problem had a simple structure and did not depend too much on the images” [triangle]; “The image was nice” [pigs in a pen]. ***Examples (least enjoyable problems)***: “It was too stylized ” [deer problem]; “The elements in the image depended too much on the overall configuration” [five square].

### Materials and methods

#### Participants

Two hundred and sixteen Italian undergraduate students (101 males, 115 females, *M* = 21.9 years, *SD* = 6.97 years) participated in the study (72 in the 7 min condition, 72 in the 3 min condition, 72 in the no engagement condition). The experiment was carried out in a room at the University of Macerata, Italy. All of the participants gave their written informed consent. The study conforms to the ethical principles of the declaration of Helsinki (World Medical Association, 2013) and was approved by the ethical committees of the University Departments of the researchers involved in study.

#### Materials

**Figure 1:**
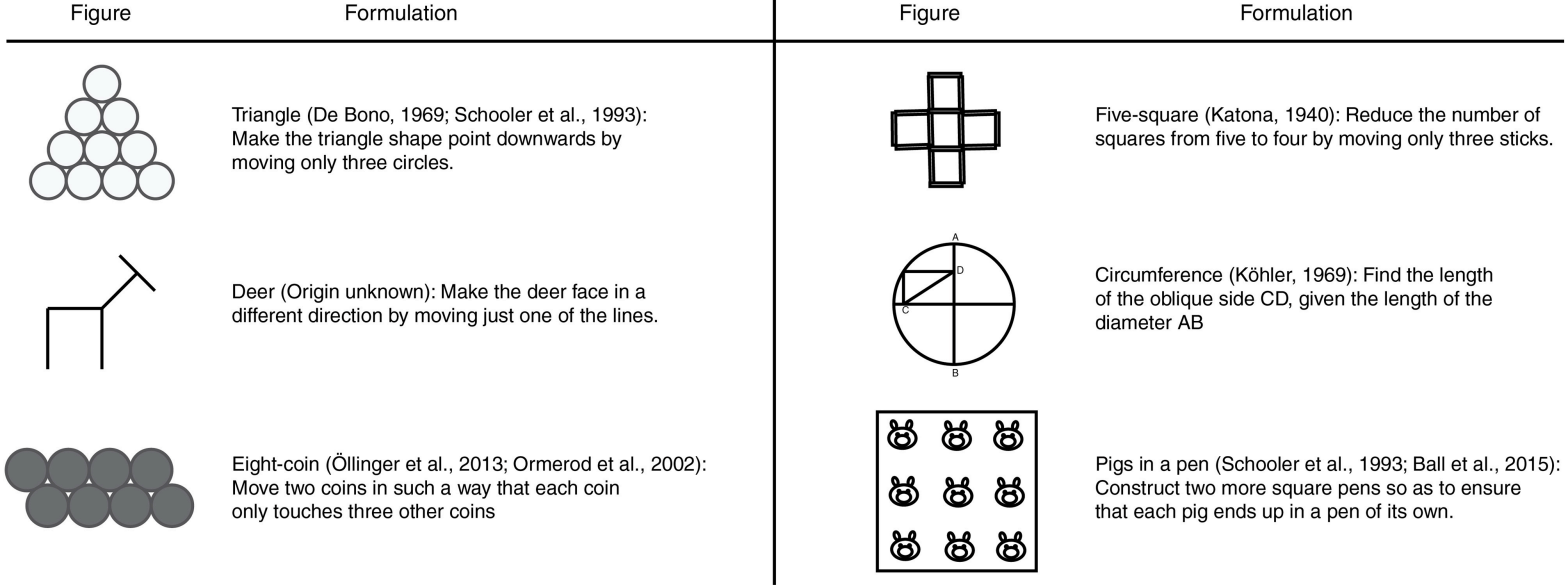
The problems used in Study 1.

Six visuo- spatial insight problems were used in all conditions (see Figure 1). The order of the six problems was randomized between participants.

#### Procedure

One booklet was given to each individual participant with the six problems printed on separate A4 sheets of paper (with the order randomized between individuals). The instructions were read out by the experimenter and projected on a screen. In the two engagement conditions (i.e., 7 min engagement and 3 min engagement), the participants were given 7 and 3 min, respectively, to read and solve each problem. They were instructed to raise their hands when they thought they had found the correct solution. If the solution was correct, they could stop, if not, they were encouraged by the experimenter to keep searching until the end of the time at their disposal. After participants had tried to solve all six problems, they were given a sheet of paper showing a table with the title of each problem, its solution and a brief explanation of the solution (solution sheet). In the third condition, no engagement, participants were simply given the initial booklet and then immediately afterwards the solution sheet.

In all three conditions, the participants were then requested to specify on a preference sheet the two problems that they considered to be the most enjoyable and the two that they considered to be the least enjoyable. In both cases, they were also asked to explain their choices in an open-answer format. There were no time limits to this last phase, but all of the participants completed the task within 15 min. The language used in the task was Italian.

#### Categorization of responses

Responses were analyzed based on the six different cognitive emotions described in the TPM (see Table 1) with three other categories (i.e., Happiness, Content type, Superficial aspects) which were added after an initial inspection of responses in order to exhaustively cover all the types of reasons referred to by the participants in the study.

Responses were classified by two independent judges with reference to each of the nine categories. Binomial coding was used, that is, the values 1 or 0 were assigned to each of the nine categories based on whether they were included in the responses or not. Each *response* (as a whole) was assigned to *at least* one category. However, it was also possible to assign it to more than one category depending on how many “chunks” (pieces of information) it could be divided into. For example, the response stating: “I found the end totally unexpected and I also liked the caricature of the faces of the subjects” was divided into two chunks since the first part refers to a violation of expectation and the second part to the superficial aspects of the cartoon (i.e., a different category). Each *chunk* was assigned to only one category. The categories that we used, technically, are partitions in that none of the categories is empty, and all the categories are disjoint sets. Both judges classified all of the responses. The inter-rater agreement was very good (*Cohen's* κ = 0.901, *SE* = 0.043). In the very few cases where the initial classifications done by the two judges did not match, a discussion took place with a third judge, and a final agreement was always reached.

#### Statistical analyses

Responses were analyzed using Mixed-effect Models (Bates et al., 2015) which make it possible to deal with the variability of some factors as random effects and with the variability of other factors as fixed effects. In all the analyses, Subjects and Problems constituted random effects. In particular, we used Generalized Linear Mixed effects Models (GLMM) with the logit link function and binomial family in the case of proportions and the Poisson family in case of counts^1^. All analyses were carried out using the statistical software program R 3.3.1, with the “lme4” (Bates et al., 2015), “lsmeans” (Lenth, 2016), and “effects” (Fox, 2003) packages. We performed Mixed Model ANOVA Tables (Type 3 tests) via Wald chi-square tests implemented in the “car” package (Fox and Weisberg, 2011). Bonferroni corrections were applied to *post-hoc* comparisons. Frequency Bubble Plot were made with the “ggplot2” package (Wickham, 2016).

### Results

**Figure 2:**
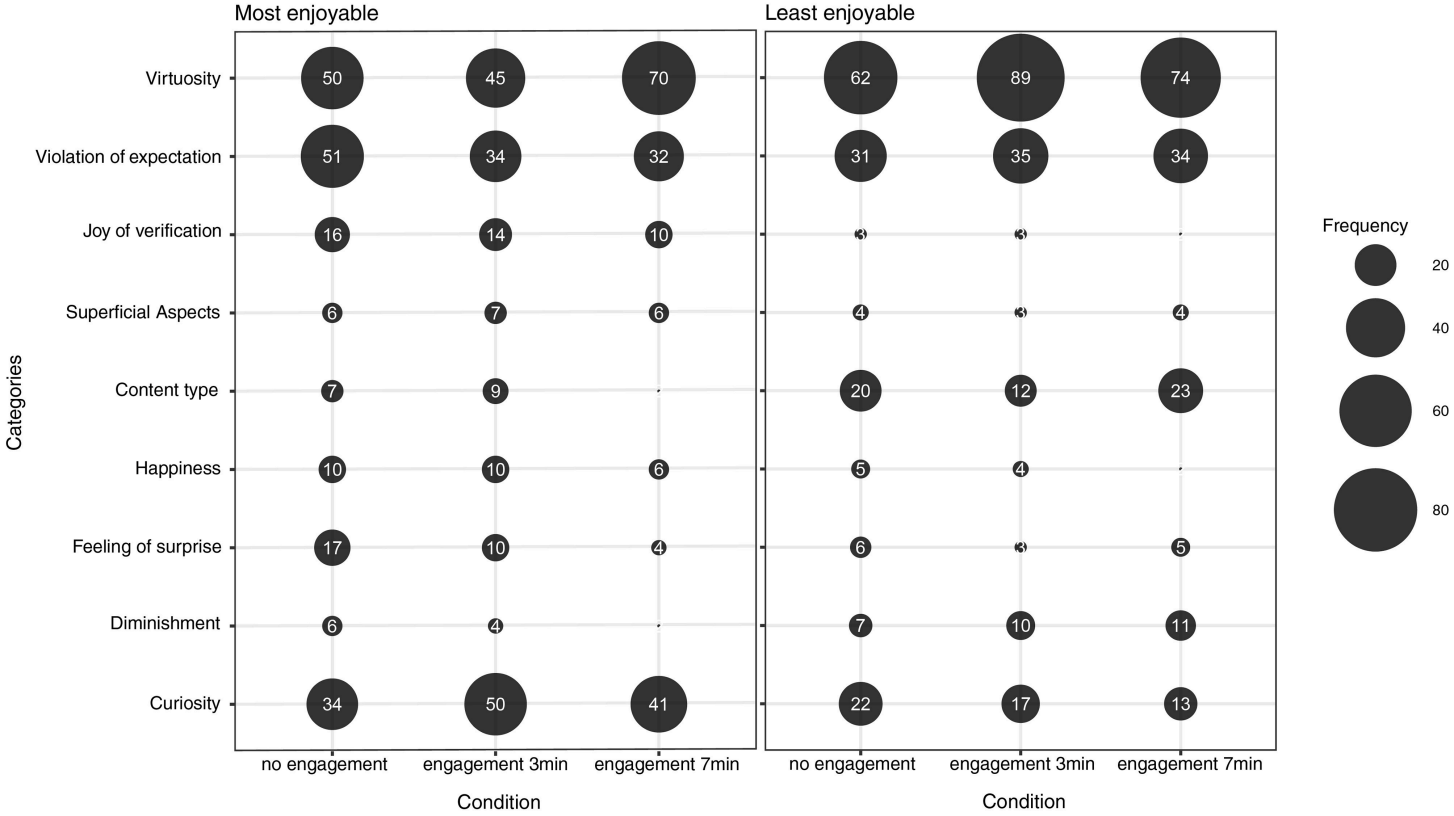
Frequency Bubble Plots showing the overall frequency of the various Categories reported by participants in relation to their choices of the most enjoyable (graph on the left) and least enjoyable (graph on the right) problems.

The bubble plots shown in Figure 2 provide a first indication of the overall frequency of the various types of explanation which the participants gave for their choice of most enjoyable or least enjoyable problems. As the plot on the left indicates, the explanations that were mentioned most frequently concerned Virtuosity, Violation of expectations and Curiosity. All of these three categories were also frequently used to explain why some problems were considered to be less enjoyable (bubble plot on the right in Figure 2), with the addition of considerations concerning feelings of happiness deriving from the activity of insight problem solving processes (Content type).

**Figure 3:**
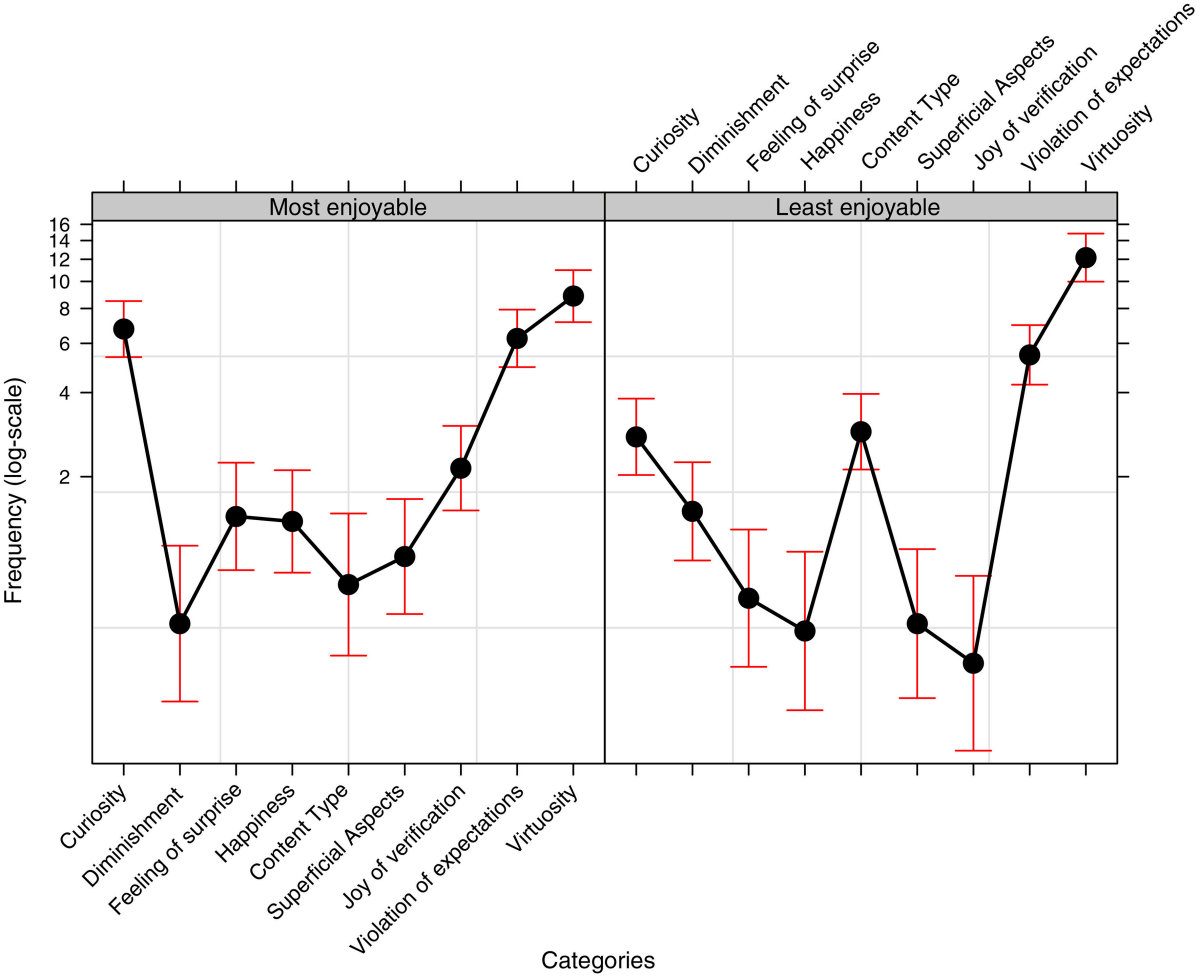
Effect plot of the frequency data (with reference to the binomial model described in the main text) showing the proportional use (use over nonuse) of the various Motivation Categories relating to the participants' choices of the most and least enjoyable problems. Bars represent a 95% confidence interval.

A GLMM (binomial, logit-link function, with Category, Condition and Enjoyability as Fixed effects) was conducted to test how responses were distributed in the three conditions, in relation to the two levels of Enjoyability (most and least enjoyable). This was done after the variability relating to the two random factors had been isolated (Subjects and Problems as random effects). The results are shown in Figure 3 and summarized in Table 2.

**Table 2:** Summary of the significant *post-hoc* tests resulting from the GLMM carried out on the explanations provided by participants to support their choices of the two most enjoyable and the two least enjoyable insight problems.

***Post-hoc* pairwise contrasts**	***z*-test**	***p*-value**	**Standard error**	**Effect size (odds ratio)**
Curiosity in the most enjoyable problems > Curiosity in the least enjoyable problems	5.689	<0.001	0.431	2.457
Joy of verification in the most enjoyable problems > Joy of verification in the least enjoyable problems	4.345	0.002	0.073	0.199
Content type in the least enjoyable problems > Content type in the most enjoyable problems	4.134	0.005	1.086	3.548
Curiosity in the most enjoyable problems (3 min condition) > Curiosity in the least enjoyable problems (3 min condition)	4.325	0.021	0.688	2.976
Curiosity in the most enjoyable problems (7 min condition) > Curiosity in the least enjoyable problems (7 min condition)	4.300	0.024	0.740	3.185
Virtuosity in the most enjoyable problems (7 min condition) > Virtuosity in the most enjoyable problems (no engagement condition)	4.430	0.013	0.580	2.573
Curiosity in the most enjoyable problems (3 min condition) > Virtuosity in the most enjoyable problems (no engagement condition)	4.307	0.023	0.638	2.751
Virtuosity in the least enjoyable problems (3 min condition) > Virtuosity in the least enjoyable problems (no engagement condition)	4.938	<0.001	0.532	2.629

The interaction between Category and Enjoyability turned out to be significant [$\chi_{\left(8,\;\text{N}\;=\;216\right)}^{2}$ = 32.742, *p* ≤ 0.001], which indicates that the frequency of the various Categories significantly differed for the most enjoyable vs. the least enjoyable problems. As *post-hoc* tests revealed:

- significant differences emerged for only three Categories. Curiosity and Joy of verification were used more often in relation to the two most enjoyable as compared to the two least enjoyable problems. This means that being curious and searching for a solution, as well as discovering that the solution was correct (or nearly correct) triggered pleasurable sensations.
- feelings of Happiness relating to the specific kind of cognitive task (Content type) were conversely proportionally more frequent in the case of explanations for the choice of the least enjoyable problems as compared to the most enjoyable problems. In this case, the category was obviously used negatively (e.g., “I've never liked geometrical problems….,” see some of the examples provided in Table 1). This suggests that unpleasant feelings were linked more to an *a priori* negative evaluation of the type of task than to any specific difficulties encountered.

A significant interaction involving Category, Enjoyability and Condition also emerged [$\chi_{\left(16,\;\text{N}\;=\;216\right)}^{2}$ = 38.442, *p* < 0.001], while the interaction between Category and Condition did not turn out to be significant [$\chi_{\left(16,\;\text{N}=216\right)}^{2}$ = 17.445, *p* = 0.357]. This latter result indicates that the three conditions did not lead, *per se*, to a different frequency with regard to the various Categories. Conversely, as the former finding indicates, differences only emerged between Category and Condition in interaction with the Enjoyability factor. Indeed, as *post-hoc* tests confirmed:

- in both of the engagement conditions, the participants referred to Curiosity more often in relation to the problems they liked the most as compared to those they liked the least;
- Virtuosity was mentioned more often as an explanation for choosing the two most enjoyable problems in the 7 min engagement condition than in the no engagement condition; (This result is consistent with the fact that feelings of virtuosity can only be experienced at the end of a successful search process. Learning the right strategy obviously implies engaging in the search for a reasonable amount of time, that is, sufficient time for the participant to feel virtuous. Those who did not engage in the search phase were obviously not in a position to feel virtuous);
- Curiosity was given as the explanation for the choice of the two most enjoyable problems more often by those participants who had been engaged in the search phase for only 3 min as compared to those participants who had not been engaged at all;
- a difference between being engaged in the search phase for only 3 min and not being engaged at all also emerged with respect to Virtuosity in the participants' explanations for their choice of the least enjoyable problems.

These findings suggest that participants in the 3 min condition were able to start the search phase and thus experience the typical emotions characterizing the early stages of problem solving (which are related to Curiosity). However, they could not experience the emotion characterizing the final phase, that is virtuosity, since they did not have sufficient time to find the correct solution. In fact, in the 3 min condition, Virtuosity was more frequently mentioned by participants in a negative sense, that is, they did not find the problem enjoyable since they did not have time to find the correct solution and therefore did not feel virtuous.

In a further analysis, we investigated whether the explanations given for both the most and least enjoyable problems changed depending on whether participants succeeded or not in finding the correct solution. To do this, we zoomed in on the two conditions in which the participants had been engaged in a search phase (7 min condition, and 3 min condition) and studied whether the frequency of the various Categories varied depended on whether or not they had been able to solve the problems. We conducted two new GLMMs (binomial family, logit link function), one to study the effects of Category (on 9 levels), Condition (3 min engagement, and 7 min engagement), and Success (problem solved correctly, problem not solved correctly) in relation to the two most enjoyable problems and another in relation to the least enjoyable problems. In both cases, a significant interaction between Category and Success emerged. The results are summarized in Table 3.

**Table 3:** Summary of the significant *post-hoc* tests resulting from the two GLMMs conducted (one on the two most enjoyable problems, another on the two least enjoyable problems) to study the effect on the explanation category of having solved or not solved the problem.

***Post-hoc* pairwise contrasts**	***z*-test**	***p*-value**	**Standard error**	**Effect size (odds ratio)**
Violation of expectations in the most enjoyable problems (problem not solved correctly > problem solved correctly)	4.544	0.008	1.454	4.436
Violation of expectations in the least enjoyable problems (problem not solved correctly > problem solved correctly)	5.180	<0.001	8.613	23.863
Virtuosity in the least enjoyable problems (problem not solved correctly > problem solved correctly)	6.468	<0.001	9.468	26.566
Curiosity in the least enjoyable problems (problem not solved correctly > problem solved correctly)	3.990	0.010	7.613	13.993
Content type in the least enjoyable problems (problem not solved correctly > problem solved correctly)	3.498	0.071	4.142	7.293

In the case of the two most enjoyable problems [$\chi_{\left(8,\text{N}\;=\;144\right)}^{2}$ = 18.780, *p* = 0.016], the difference concerned the Violation of expectations category that was more frequently mentioned in relation to unsolved problems. The fact that this category was frequently mentioned by participants in relation to problems that they enjoyed but had not been able to solve, indicates that realizing that a switch in perspective was needed (even though this only became evident when the participants' response sheets were examined) elicited pleasurable emotions. In other words, people find pleasure in discovering that a change in the initial expectations is needed to find the solution, that fixating on the initial representation of the problem causes a block and that they can overcome this block by violating the initial expectations. “Unexpected” in this case means “enjoyable.”

The interaction between Category and Success was also significant in the second GLMM [χ^2^_(8,*N* = 144)_ = 21.264, *p* = 0.008] which focused on the problems which were chosen as the least enjoyable (see the section on the right in Table 3). Three categories were most frequently used in association with unsolved problems: Violation of expectations; Virtuosity and Curiosity. A tendency also emerged in the case of Content type. These results indicate that participants who had not being able to solve a problem and evaluated it as unpleasant/ not enjoyable reported that their negative feeling related to not having experienced being skilled enough to succeed in finding the correct solution (i.e., lack of Virtuosity), or not having felt stimulated by the problem (i.e., no Curiosity), or their frustration at not having being able to change their initial perspective (i.e., Violation of expectations).

## Study 2: factors determining enjoyment or lack of enjoyment in humor

The results from **Study 1** showed which categories (in terms of the TPM) occurred the most frequently in the participants' explanations for their choice of the most and least enjoyable visuo-spatial insight problems of the six that they worked on. In this second study, again using the TPM as a point of reference, we aimed to explore the categories that were the most frequently included in the explanations given by the participants for their choice of the most and least enjoyable of the six captioned cartoons they were shown. In caption cartoons (also called mixed mode cartoons), both the pictorial and the textual aspects are pivotal to the interpretation of their humorous interpretation (Attardo and Chabanne, 1992; Tsakona, 2009). The reason for choosing this type of cartoon for the second study as compared to, for instance, verbal jokes, was that the six visual-insight problems used in Study 1 were also mixed mode since they consisted of both drawings and verbal texts.

Humorous stimuli are supposed to be understood quickly, otherwise the humorous effect diminishes or fails (Derks et al., 1998; Cunningham and Derks, 2005). For this reason, it was not possible to test different time conditions in Study 2 as in Study 1. The process of understanding humor is immediately activated by the presentation of a stimulus. We modulated the immediacy of the participant's access to the punch line by using one-panel and multi-panel versions of the same cartoons but the times involved were still very short. In visuo-spatial insight problems, the initial representation is provided together with a text describing the task, while the representation displaying the solution is shown at a later point (unless the problem solver immediately sees the solution but this is extremely rare). In one-panel cartoons, all the information is condensed into one image. In multi-panel cartoons, the information (i.e., the onset and resolution) is distributed across the panels and the resolution is only displayed in the last one. In this sense, spreading out the participant's access to the initial and to the final parts of the joke is more similar to what normally happens in problem solving tasks, although within a much longer timeframe.

### Materials and methods

#### Participants

One hundred and eighty four Italian undergraduate students (96 males, 88 females, *M* = 21.8 years, *SD* = 6.44 years) participated in the study (86 in the multi-panel condition, 98 in the single-panel condition). The experiment was carried out in a classroom at the University of Verona (Italy) at the end of a class which was totally unrelated to the topic of the study. All of the participants gave their written informed consent. The study conforms to the ethical principles of the declaration of Helsinki (World Medical Association, 2013) and was approved by the ethical committees of the University Departments of the researchers involved in study.

#### Materials

**Figure 4:**
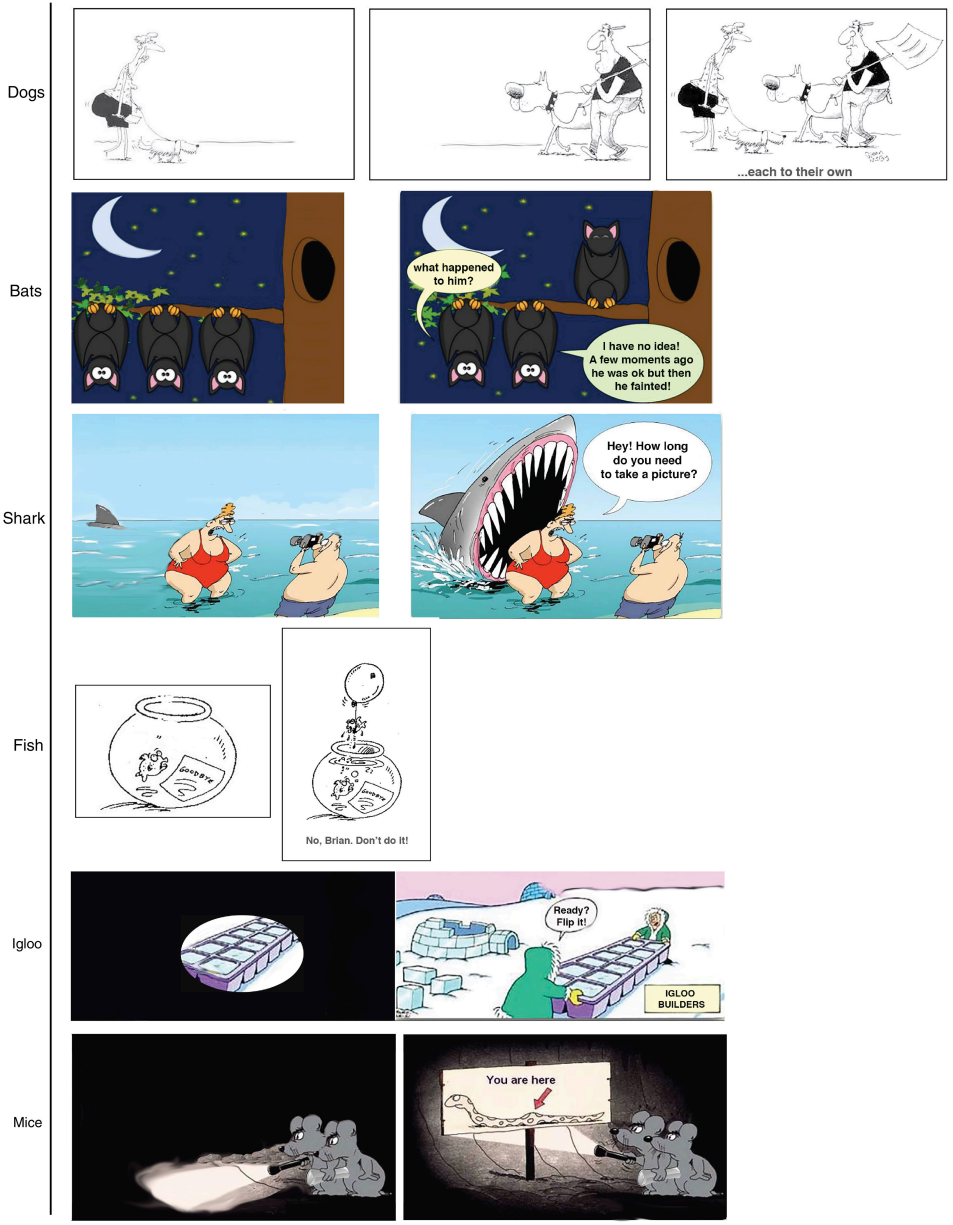
The cartoons used in Study 2 as presented in the multi-panel condition. In the one-panel condition, only the final panel (i.e., the one on the right) was presented. The original versions of the cartoons (one-panel, in Italian) were retrieved from www.paginainizio.com on the 15th September, 2017 (by courtesy of PaginaInizio.com).

Six caption cartoons were used. The cartoons had been taken from a website on the internet. All of them were one-panel cartoons but we modified them in order to obtain an additional multi-panel version (see Figure 4).

#### Procedure

One booklet containing the 6 cartoons was given to each individual participant with the order of the cartoons randomized between participants. The cartoons were all one-panel cartoons in one condition and all multi-panel cartoons in another condition.

The instructions were read out by the experimenter and projected on a screen. Participants were asked to look at and read the six cartoons. A sheet of paper containing a brief explanation for each cartoon was then provided (paralleling the solution sheet in Study 1). It was felt that this was needed to guarantee that everyone understood the jokes. The participants were then requested to specify which two cartoons they considered to be the most enjoyable and which two they considered to be the least enjoyable. They were also asked to explain their choices (open-answer). The format of the response sheet was identical to the one used in Study 1 with a space for them to indicate their choices and five lines for each choice in which they were requested to explain what made the cartoon particularly enjoyable or otherwise. There were no time limits, but all of the participants completed the task within 10 min. The language used for the task was Italian.

#### Categorization of responses

Responses were analyzed with reference to the set of categories used in Study 1 (see Table 1) adapted for use with the cartoons (see Table 4). For the sake of simplicity, the cartoons are referred to as jokes since traditionally cartoons are frequently visual jokes (Attardo and Chabanne, 1992; Corcoran et al., 1997; Hempelmann and Samson, 2008). The application of this set of categories to humor was done on the basis of the TPM and of an initial inspection of the responses in order to guarantee that the operative tools used represented the complexity of the qualitative explanations of the participants. All of the responses were classified by two independent judges in terms of each of the nine categories. Binomial coding was used, that is, the values 1 or 0 were assigned to each of the nine categories based on whether they were contained in the responses or not. The categories were therefore not mutually exclusive. The inter-rater agreement was very good (*Cohen's* κ = 0.879, *SE* = 0.051). In the very few cases where the initial classifications done by the two judges did not match, a discussion took place with a third judge, and a final agreement was always reached.

**Table 4:** Operational categories used to analyze the explanations provided by participants in Study 2.

Curiosity	***Definition in Humor:*** Curiosity is associated with realizing that there is something incongruous in the text that needs to be understood, and this incongruity elicits a state of tension which activates the person to look for meanings. Once the resolution of a joke is achieved, a positive feeling of knowing something new (i.e., getting the joke) arises and this leads to a final state of relief. ***Examples (most enjoyable cartoons)***: “It stimulated my curiosity and made me want to understand the meaning” [bats]; “It made me curious and it interested me; this pushed me to look more carefully at the snake…it was clear that there was something humorous hidden somewhere…” [mice]. ***Examples (least enjoyable cartoons):*** “From the beginning, it left me indifferent and did not stimulate any reaction in me or any curiosity [dogs]”; “It did not catch my attention, and therefore it did not make me want to understand its meaning” [igloo].
Virtuosity	***Definition in Humor:*** Virtuosity is the feeling of being able to understand a joke. It occurs more frequently when the joke is perceived as witty or is based on intellectual or specific domain knowledge than when it is perceived as trivial and can be understood by everyone. ***Examples (most enjoyable cartoons)***: “It was ironic to the right point: you needed to think about it a bit” [igloo]; “It offers a brilliant comparison; it is not immediately clear” [igloo]. ***Examples (least enjoyable cartoons):*** “It is much too convoluted and complicated, and did not make me laugh immediately” [bats]*;* “I was not able to understand immediately what the point of the joke was; I focused on an irrelevant aspect…” [dogs].
Violation of expectations	***Definition in Humor:*** Violation of expectations is related to discovering that the joke plays on contravening/contrasting expectations toward which the reader has been biased by the text/image at the beginning of the interpretative process, and to discovering that the resolution of the joke requires a re-structuring of the initial interpretation. ***Examples (most enjoyable cartoons):*** “It is paradoxical. Exactly the opposite of what is true for humans!” [bats]; “It is uncommon and unusual, and it excited my interest precisely because it is absurd” [mice]. ***Examples (least enjoyable cartoons):*** “It did not amuse me because the cartoon is too far from reality, it is unreal, absurd.” [fish]; “I did not enjoy the igloo cartoon because the solution is too far from what actually happens” [igloo].
Feeling of surprise	***Definition in Humor:*** Feelings of surprise arise when a new interpretation, which is achieved by resolving an incongruity, is perceived as unusual. ***Examples (most enjoyable cartoons):*** “The punch line was unexpected” [fish]*;*“The punch line surprised me” [mice]. ***Examples (least enjoyable cartoons)**:* “It is banal and predictable” [shark]; “The end was predictable” [fish].
Joy of verification	***Definition in Humor:*** Joy of verification is experienced depending on the proximity of a person's understanding to the correct interpretation of the joke. (note: this category differs from Virtuosity in that the participants did not explicitly refer to their ability to understand quickly or to the subtlety of the jokes but rather mentioned the outcome of their understanding matching the “official”interpretation of the joke). ***Examples (most enjoyable cartoons):*** “It is an easy and immediate punch line, it does not require reasoning and you understand it quickly” [shark]; “I got it immediately and this made it very humorous” [igloo]. ***Examples (least enjoyable cartoons):*** “I could not get the meaning from the drawing and the text” [dogs]*;* “If I had not been given the explanation of the joke, I would have never have thought it was meant to make people laugh” [dogs].
Diminishment	***Definition in Humor:*** Diminishment is experienced when the reinterpretation of the text (i.e., the resolution of the incongruity) implies that the characters or the event on which the joke is focused are less attractive (e.g., honest, innocent, loyal, clever) than they seemed from the first impression. ***Examples (most enjoyable cartoons):*** “It is amusing that the little mice have not realized before that they have been eaten”*;* “The husband, with the excuse of taking a picture of his wife, tries to give the shark time to eat his wife, who apparently has no idea what is happening [shark]. ***Examples (least enjoyable cartoon):*** “It plays on the perceived stupidity of mice” [mice]*;* “I was sorry for the mice and felt bad when I realized that they had understood that they were in a snake's belly.” [mice]
Happiness	***Definition in Humor:*** a pure expression of amusement (i.e., appreciation of a joke), without any specific explanation for its cause. ***Examples (most enjoyable cartoons):*** “The idea of a fish flying tied to a balloon makes me laugh.” [fish]*;* “It was ironic and playful” [shark]. ***Examples (least enjoyable cartoons):*** “There is nothing amusing about this” [dogs]; “I did not enjoy it for no specific reason but I simply find it not very ironic” [mice].
Content type	***Definition in Humor:*** an expression of appreciation and amusement connected to a specific humorous genre or humorous topic. ***Examples (most enjoyable cartoons):*** “The stereotypical topic of an annoying wife who exasperates her husband is always humorous” [shark]; “It made me laugh because it plays on the customary parody of wife and husband. The relationship between the two is often compared to the formula “love-hate relationship.” [shark] ***Examples (least enjoyable cartoons):*** “It represents typical masculine humor that is based on the idea that you need to get rid of the no longer desired wife, without caring about her general wellbeing. Male chauvinism.” [shark] ; “It is not amusing because it relates to the issue of suicide, so I think it is black humor” [fish]
Superficial aspects	***Definition in Humor:*** An expression of amusement and appreciation related to the superficial and formal aspects of a joke. ***Examples (most enjoyable cartoons):*** “I enjoyed the characters and the facial expressions used to convey the humorous meaning” [fish]; “I found the caricature of the characters funny” [shark]. ***Examples (least enjoyable cartoons):*** “I did not appreciate it mostly because of the style of the drawing” [shark]; “The characters in the cartoon are animals which I do not like” [mice].

#### Statistical analyses

Responses were analyzed using Mixed-effect Models (using the same packages as those described in Study 1). In all the analyses, Subjects and Cartoons constituted random effects. We used Generalized Linear Mixed effects Models (GLMM) with the logit link function and binomial family in the case of proportions and the Poisson family in case of counts. Bonferroni corrections were applied to *post-hoc* comparisons.

### Results

**Figure 5:**
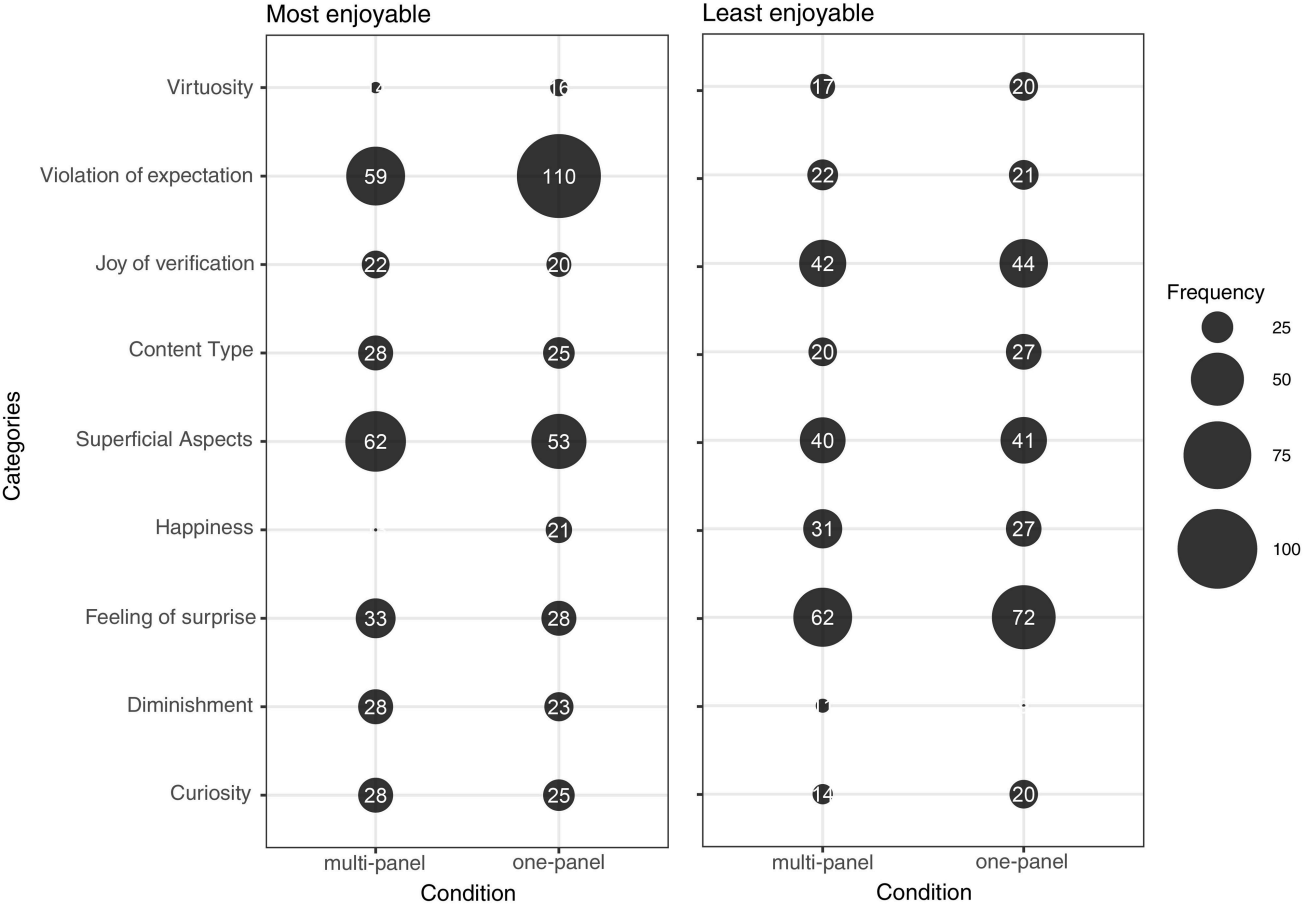
Frequency Bubble Plots showing the overall frequency of the various Categories reported by participants in relation to their choices of the most enjoyable (graph on the left) and least enjoyable (graph on the right) cartoons.

The bubble plots in Figure 5 show the overall frequency of the various types of reasons which the participants gave for their choices of the most and least enjoyable cartoons. The plot on the left suggests that Violation of expectations is often referred to, and that Structural aspects concerning the subject or the graphics of the cartoon (Superficial aspects) are also frequently mentioned. Conversely, there was a greater range of reasons given for lack of enjoyability but lack of a Feeling of surprise, lack of autonomous understanding of the joke (Joy of verification) and again specific aspects relating to the subject or graphic aspect of the cartoon (Superficial aspects) were overall the Categories that the participants most frequently referred to.

A GLMM was conducted to test how responses were distributed (binomial, logit-link function, with Category, Condition and Enjoyability as Fixed effects, Subjects and Cartoons as random effects).

**Figure 6:**
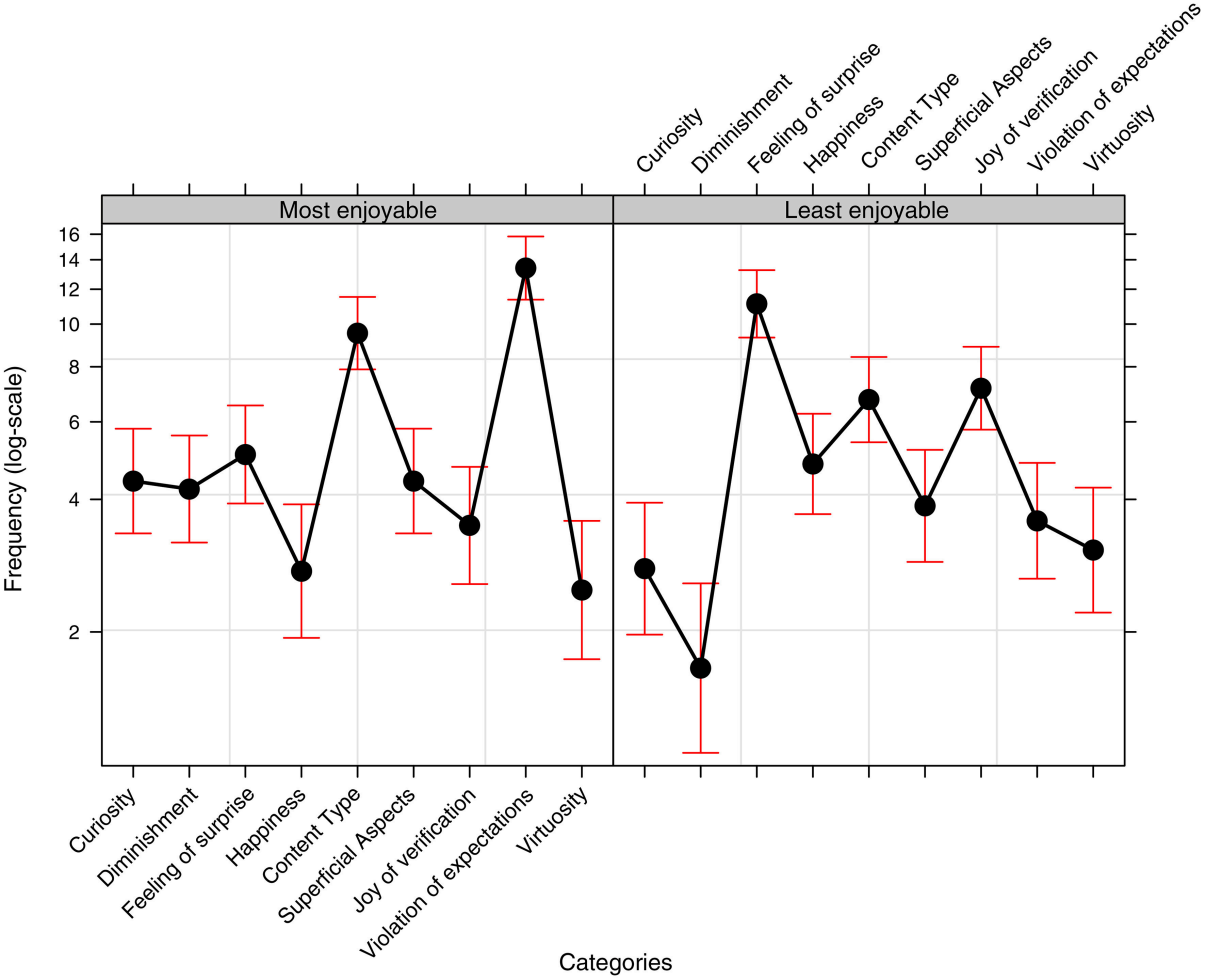
Effect plot of the frequency data (with reference to the binomial model described in the main text) showing the proportional use (use over nonuse) of the various Categories relating to the participants' choices of the most and least enjoyable cartoons. Bars represent a 95% confidence interval.

As the significant main effect of Categories indicates [χ^2^_(8,*N* = 184)_ = 82.803, *p* < 0.001], some of the Categories were more frequently used by participants to explain their choices than others. In particular (as *post-hoc* tests confirmed), Feeling of surprise, Violation of expectations and aspects relating to the content or graphics of the cartoons (Superficial aspects) were the three categories most frequently used. However, the interaction between Category and Enjoyability also turned out to be significant [$\chi_{\left(8,\text{N}=184\right)}^{2}$ = 68.111, *p* < 0.001], which means that the frequency of the various Categories significantly differed for the most enjoyable vs. the least enjoyable cartoons. In fact, as shown in Figure 6 (and confirmed by the *post-hoc* tests reported in Table 5), Violation of expectations and Diminishment were used more often in relation to the two most enjoyable as compared to the two least enjoyable cartoons. Conversely, lack of Feeling of surprise and the absence of Joy of verification were more frequently referred to when explaining the choice of two least enjoyable cartoons. References to structural aspects were used with the same frequency for both the most enjoyable and the least enjoyable cartoons (*Odds-ratio* = 0.618, *SE* = 0. 041, *z-ratio* = 2.831, *p* < 0.708).

**Table 5:** Summary of the significant *post-hoc* tests resulting from the GLMM carried out on the explanations provided by participants to support their choices of the two most enjoyable and the two least enjoyable cartoons.

***Post-hoc* pairwise contrasts**	***z*-test**	***p*-value**	**Standard error**	**Effect size (odds ratio)**
Violation of expectation in the most enjoyable cartoons > Violation of expectation in the least enjoyable cartoons	9.315	<0.001	0.662	6.173
Diminishment in the most enjoyable cartoons > Diminishment in the least enjoyable cartoons	3.732	0.029	0.752	2.809
Feeling of surprise in the least enjoyable cartoons > Feeling of surprise in the most enjoyable cartoons	5.926	<0.001	0.511	2.872
Joy of verification in the least enjoyable cartoons > Joy of verification in the most enjoyable cartoons	4.188	<0.001	0.486	2.365

Therefore, the findings which emerged from this study suggest that understanding a cartoon (Joy of verification) and being surprised by it (Feeling of surprise) are two conditions which are essential for pleasure: when they were not present, the cartoon was not perceived as being enjoyable. At the same time, being surprised by the punch line and understanding it do not seem to be enough to guarantee a greater degree of enjoyment: recognizing a violated expectation and experiencing a diminishment in the cleverness or awareness initially attributed to the characters of the joke were the two aspects which were specifically more frequently associated with the most enjoyable cartoons.

No significant differences in the distribution of responses in the one-panel as compared to the multi-panel condition emerged, there was only a trend [$\chi_{\left(8,\text{N}=184\right)}^{2}$ = 14.701, *p* < 0.065]. A *post-hoc* inspection revealed that this related to a relatively lower frequency of the Category entitled Violation of expectations as a reason for a cartoon being chosen as the most enjoyable in the multi-panel condition (*Odds-ratio* = 0.408, *SE* = 0.088, *z-ratio* = −4.146, *p* = 0.021). We will go back to this finding in the final discussion.

## Discussion

In this study, we explored whether new elements relating to the enjoyment experienced in problem solving and understanding humor might be discovered by comparing these two cognitive activities within a general theory of the Pleasures of the Mind. The theory we assumed as a framework (Kubovy, 1999) is based on the idea that all pleasures of the mind derive from a narrative structure which activates a corresponding sequence of emotions. The concept of narrative interpretation applies equally well to the processing involved in both solving an insight problem and understanding a joke. In two studies (one focusing on visuo-spatial insight problems, and the other on cartoons), we explored the applicability of the same set of categories in order to analyze the participants' choices of the most enjoyable or least enjoyable problems and cartoons. We do not wish to imply that these categories describe exactly the same aspect in the two contexts. Every time general categories are instantiated in different areas (and even in different individual cases within the same area, e.g., in our case, in specific cartoons or specific visuo-spatial problems), their meaning changes slightly. There is, however, still an element which is invariant. Tables 1, 4 show how we modulated the same general categories for the purposes of the two contexts. Table 1 applies to visuo-spatial insight problem solving and Table 4 to humor. The interpretations do not aspire to be definitive; rather, they represent an initial operational proposal derived from the general definitions provided by Kubovy (1999). The question was whether putting both activities under a common umbrella (as suggested by the TPM) might reveal something in common in terms of the relative underlying cognitive mechanisms. At the present state of the art, it was not possible to formulate a predictive hypothesis regarding the application of the abovementioned set of categories to two different cognitive activities. In fact, in the original paper (Kubovy, 1999), the application of the TPM to humor and problem solving was more hinted at than actually demonstrated analytically.

With all these premises in mind, we still consider the results of our research to be extremely encouraging and further testing would certainly be worthwhile. An evaluation of whether the results of the studies also offer useful feedback in terms of a theoretical elaboration of the theory which was assumed as a framework, that is the TPM, is beyond the scope of this paper. In this paper, we have shown that the mindset underlying the TPM supports the idea of re-conceptualizing many of the proposals which have been developed in research on the subject of problem solving and humor (sections Connections between the TPM Approach and More “Local” Theories Relating to the Emotions Elicited by Insight Problem Solving and Connections between the TPM Approach and More “Local” Theories on Humor). Furthermore, we have shown that a joint application of this set of common terms to both visuo-spatial insight problems (section Study 1: Factors Determining Enjoyment and Lack of Enjoyment in Insight Problem Solving) and cartoons (section Study 2: The Factors Determining Enjoyment or Lack of Enjoyment in Humor) revealed a varying prominence of the various categories.

In problem solving, Curiosity and Joy of verification were the most often referred to in relation to the problems which were judged to be more enjoyable. This means that being fascinated by a problem and then happy to discover that the solution is in fact correct (or nearly correct) both trigger a “pleasure of the mind” experience. Conversely, lack of enjoyment was more frequently linked to an a priori negative evaluation of the type of task (category Content type) than to any specific difficulties which had been encountered during the search phase. By investigating three conditions, two requiring the participants to engage in a search for a solution (i.e., 3 min engagement or 7 min engagement) and one requiring them to simply read the problems and their solutions, it was possible to verify that Virtuosity occurred in relation to the two most enjoyable problems significantly more frequently for those participants who had been engaged in the search for 7 min as compared to those who had not been engaged at all. Engaging in a search for the solution for a reasonable amount of time (i.e., long enough to try various strategies and therefore experience being “virtuous”) thus seems to be a critical factor in terms of whether or not the problem solver experiences pleasure related to virtuosity in this kind of task. Participants who were not engaged in the search phase obviously could not experience feelings of virtuosity. Those who were engaged in the search phase for only 3 min were able to experience the positive emotions that, according to the TPM, typically characterize the early stages of processing in an enjoyable activity, namely Curiosity, but they were unable to experience the emotions characterizing the final stages (e.g., Virtuosity). In fact, this latter category was usually chosen as a reason for lack of enjoyment due to feelings of frustration, that is, for negative rather than positive reasons. Finally, realizing that a change in perspective was needed in order for the problem to be solved (i.e., Violation of expectations) turned out to be a clear source of enjoyment for some but a clear source of lack of enjoyment for others. In fact, Violation of expectations was one of the most frequently reported explanations in association with both the most and the least enjoyable problems. In particular, it was more frequently reported as the cause of lack of enjoyment by those participants who had failed to solve the problems as compared to those who had succeeded (and the same held for lack of Virtuosity and lack of Curiosity).

With regard to the cartoons, Joy of verification and Feeling of surprise turned out to be two essential categories. Indeed, absence of understanding (or of clear understanding) and absence of surprise were the two categories which were significantly associated with the cartoons which were judged to be the least enjoyable. Violation of expectations was another category which occurred frequently but, in contrast with the results of the problem solving study, it was only specifically mentioned as a reason for enjoyment (i.e., it was associated with the most enjoyable rather than the least enjoyable cartoons).

As things stand, it is not possible to ascertain whether the findings of our two studies are specific to the six problems and the six cartoons used or whether it is a generalizable outcome. Further studies extending the analysis to a different sample of problems and humorous stimuli would be required for this to be established. However, as already clarified, the ambition of this paper was in no way to be all inclusive or conclusive but rather to open a research path. The above findings paint a reasonable picture of the similarities and differences relating to people's experiences of pleasure of the mind resulting from these two activities. With regard to the differences, for example, the fact that Virtuosity played a major role in problem solving but not in understanding humor seems to be in line with the consideration that the incongruity which is a basic component of humor is noticed and resolved quickly in cartoons, whereas the re-organization of a problem that needs to be addressed in insight problem solving is neither fast nor without effort. This effort is part of the process and, as the responses of our participants confirmed, also part of the pleasure. In contrast, finding humor difficult to understand is not experienced as a part of the process; as one of the participants in **Study 2** clearly said “Even after I had understood the humor in the cartoon when I read the explanation, I could *understand* what the point was but I only got it in my “head”: I didn't *experience enjoyment*.”

As a final consideration, we would like to focus on the major role of Violation of expectations which emerged in both studies. In a totally different context, i.e., a cognitive analysis of the reasoning mechanisms underlying problem solving and humor, it has been demonstrated that contrast is key to any exploration of alternative strategies in insight problem solving (Branchini et al., 2015, 2016), as well as in inductive (Gale and Ball, 2012) and deductive thinking (Augustinova, 2008), and it has also been argued that contrast is fundamental to the incongruity mechanism in humor (Colston, 2002; Canestrari and Bianchi, 2012, in press; Canestrari et al., 2017; see reviews in Keith-Spiegel, 1972; Martin, 2007; Larkin-Galiñanes, 2017). The results discussed in the present paper (in particular with respect to the Violation of expectations) suggest that contrast also represents a link between insight problem solving and humor in terms of the cognitive emotions triggering pleasures of the mind.

One of the aspects that we are aware our experimental design did not factor in is the perceived complexity of the problems which were presented to the participants. In Berlyne's aesthetic theory (1972), he used the “inverted U paradigm” to demonstrate how a stimulus of medium complexity elicits an intermediate level of arousal which impacts positively on the hedonic value of the stimulus. This paradigm has also been used within the literature on humor (e.g., (Berlyne, 1972; Wyer and Collins, 1992) and the references therein) to describe the relationship between the complexity of a joke and its entertainment value, whereas, to our knowledge it has not been used to describe difficulties experienced in problem solving and the pleasure derived from it. It was also extensively discussed in Kubovy's original paper (1999). This is an aspect, in addition to widening the range of insight problems (i.e., visuo-spatial insight problems) and humor stimuli (cartoons) to include other types, that future studies would need to address.

## Ethics statement

All subjects gave written informed consent in accordance with the Declaration of Helsinki. The protocol was approved by the ethical committees of the Department of Education, Cultural Heritage and Tourism, University of Macerata, Department of Humanities, University of Macerata, Department of Human Sciences, University of Verona.

## Author contributions

CC, EB, IB, US, RB substantially contributed to the conception of the work, the design of the study, the drafting of the work, and the interpretation of the data. CC, IB, EB contributed to the acquisition of the data; CC and EB contributed to the coding of responses; RB and IB contributed to the analysis of the data. CC, IB, EB, RB, US approved the final version to be published and agree to be accountable for all aspects of the work in terms of the accuracy or integrity of any part of the study.

### Conflict of interest statement

The authors declare that the research was conducted in the absence of any commercial or financial relationships that could be construed as a potential conflict of interest.

## Footnotes

^1^Generalized Linear Mixed Models (GLMM) allowed us to deal with variability related to the items and to the subjects as a random effect. The items used in the experiment were in fact simply exemplars of visuo-spatial geometrical problems and humorous captioned cartoons and—in our experimental design—they were interchangeable with other items of the same type. Fixed effects are constant across individuals and random effects vary (Kreft and De Leeuw, 1998). Fixed effects are interesting in themselves; effects are random if the focus of interest in on the underlying population (Searle et al., 2008; Snijders and Bosker, 2011). Link-function refers to the link between factors/covariates and responses. It explains how the expected value of the response relates to the linear predictor of explanatory variables. Linear regression assumes that the response variable is normally distributed (Dobson and Barnett, 2008). GLMM can have response variables with distributions other than the Normal distribution—they may even be categorical rather than continuous. Thus they may not range from – ∞ to + ∞; the relationship between the response and explanatory variables does not need to be in a simple linear form. This is why we need the link function: it links the mean of the dependent variable to the linear term in such a way that the range of the non-linearly transformed mean ranges from – ∞ to + ∞. Thus we can actually form a linear equation and use an iteratively re-weighted least squares method for a maximum likelihood estimation of the model parameters. Our dependent variable was coded as binomial-data: in this case link function is logit-function.
